# Supplementary material for: Learning curve and surgical outcome of robotic assisted colorectal surgery with ERAS program
Source: Sci Rep. 2022 Nov 29;12:20566. doi: 10.1038/s41598-022-24665-w (PMC9709162; doi:10.1038/s41598-022-24665-w)
Supplement: Supplementary file 2 — Supplementary Information 2. [file 41598_2022_24665_MOESM2_ESM.zip › RAL Raw data-Ñ[▒K/124/Patho_ñw╝╨░O▒Kñσ.pdf]

臺中榮民總醫院 臺中市臺灣大道四段1650號 病理部活體組織切片 臨床醫學實驗及病理診斷中心 檢驗(查)報告

[redacted]

Pathologic diagnosis:
1. Sigmoid colon, robotic assist anterior resection --- Status post polypectomy with no residual tumor. The bilateral cut ends and mesocolic lymph nodes (0/16) are free.
2. IMA root, dissection --- Unremarkable lymph nodes (0/2).
3. Colon, cut end, distal, resection --- Chronic inflammation.

Prognostic and predictive factor:
1. Tumor size: Not applicable.
2. Depth of invasion: No evidence of primary tumor.
3. Histologic grade: Not applicable.
4. Angiolymphatic invasion: Not identified.
5. Perineural invasion: Not identified.
6. Tumor deposits (discontinuous extramural extension): Not identified.
7. Macroscopic tumor perforation: Not identified.
8. Surgical Margins:
a) Proximal Margin: Uninvolved.
b) Distal Margin: Uninvolved.
c) Radial or Mesenteric Margin: Uninvolved.
9. Lymph node status: Uninvolved (0/18).
10. Extranodal involvement: Not identified.
11. Tumor regression grading S/P CCRT: No known presurgical therapy.
12. Type of polyp in which invasive carcinoma arose: Not applicable.
13. Additional pathologic findings: Not identified.
14. Pathological TNM Stage: pTxN0 (According to the eighth edition, American Joint Committee on Cancer Staging Guidelines for Tumors).
15. TNM descriptors: Not applicable.

Gross description:
The specimen consists of 1) a segment of colon, 7.5 cm in length and 2.5 cm in proximal cut end circumference, 4 cm in distal circumference, and mesocolic soft tissue. There is a surgical clip and surgical tie noted on the mucosa with dye. The surgical clip is 2.8 cm to the distal margin. There is no obvious scar noted over the mucosa. On cross section, no lesion is found grossly. 2) IMA root, a piece of tan yellow soft tissue, 3.6x2.2x1 cm. 3) distal cut end, a piece of tan mucosa-covered soft tissue, 2.2x1.8x1.6 cm. Representative part for section: A) proximal cut end B) distal cut end C-G) all of mucosa marked with dye H) radial margin J) all of specimen 2 K) specimen 3. Blue ink indicated serosa; yellow ink indicated radial margin L-V) lymph nodes.
#T-59300_2 #M-09183_2 0 1105 000000
#T-C4560_2 #M-09183_2 0 1105 000000

已電子簽章

敬告：本單僅供醫師參考使用，不具任何申請效力！！
請病患妥為保管，若不慎遺失需自負個人資料外洩責任！
